# Endogenous Retroviruses (ERVs): Does RLR (RIG-I-Like Receptors)-MAVS Pathway Directly Control Senescence and Aging as a Consequence of ERV De-Repression?

**DOI:** 10.3389/fimmu.2022.917998

**Published:** 2022-06-09

**Authors:** Eros Di Giorgio, Luigi E. Xodo

**Affiliations:** Laboratory of Biochemistry, Department of Medicine, University of Udine, Udine, Italy

**Keywords:** endogenous retrovirus, dsRNA, MAVS, RLR, senescence

## Abstract

Bi-directional transcription of Human Endogenous Retroviruses (hERVs) is a common feature of autoimmunity, neurodegeneration and cancer. Higher rates of cancer incidence, neurodegeneration and autoimmunity but a lower prevalence of autoimmune diseases characterize elderly people. Although the re-expression of hERVs is commonly observed in different cellular models of senescence as a result of the loss of their epigenetic transcriptional silencing, the hERVs modulation during aging is more complex, with a peak of activation in the sixties and a decline in the nineties. What is clearly accepted, instead, is the impact of the re-activation of dormant hERV on the maintenance of stemness and tissue self-renewing properties. An innate cellular immunity system, based on the RLR-MAVS circuit, controls the degradation of dsRNAs arising from the transcription of hERV elements, similarly to what happens for the accumulation of cytoplasmic DNA leading to the activation of cGAS/STING pathway. While agonists and inhibitors of the cGAS–STING pathway are considered promising immunomodulatory molecules, the effect of the RLR-MAVS pathway on innate immunity is still largely based on correlations and not on causality. Here we review the most recent evidence regarding the activation of MDA5-RIG1-MAVS pathway as a result of hERV de-repression during aging, immunosenescence, cancer and autoimmunity. We will also deal with the epigenetic mechanisms controlling hERV repression and with the strategies that can be adopted to modulate hERV expression in a therapeutic perspective. Finally, we will discuss if the RLR-MAVS signalling pathway actively modulates physiological and pathological conditions or if it is passively activated by them.

## Introduction: Complexities and Peculiarities of HERVs

Transposable elements (TE) make up about 46% of the human genome (1). They consist in repetitive sequences which are capable to or potentially capable to actively or passively insert copies of themselves elsewhere in the genome (1). TE are classified in Class I TEs, if they are RNA retrotransposons that require reverse transcriptase activity for transposition, and Class II TEs, or DNA transposons, that require transposase enzyme for their mobilization. LINE (long interspersed nuclear elements) and SINE (short interspersed nuclear elements) are the most studied and abundant class I TEs, representing respectively 17 and 11% of human genome (2). LINE and SINE will not be discussed in this manuscript, as there are in the literature a good number of excellent reviews on the subject.

**Figure 1:**
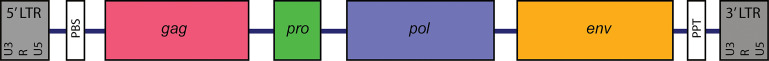
Simplified schematic structure of the organization of a complete ERV. LTR, long terminal repeat control ERV transcription and polyadenylation; *gag*, viral capsid*; pol,*reverse transcriptase*, env,* glycoprotein of the envelope*; pro,* viral protease; PBS, tRNA-specific primer-binding site; PPT, polypurine tract.

The third family of Class I TEs consists of long terminal repeat (LTR) retroelements, known as HERVs (human endogenous retroviruses) (3). HERV are residues of viral infections from the past that have remained in the human genome and occupy about 8% of it. Complete HERV elements (**Figure 1**) have a length of 1-10Kb and contains the ORF of four viral-like genes: *gag, pol, env, pro* encoding respectively the viral capsid protein, the reverse transcriptase, the envelope-associated glycoprotein and the viral protease (4). 5’ and 3’ LTR control the bidirectional transcription by RNAP2 of HERV elements (5), their polyadenylation (6) and chromatin folding (7). HERV are often not complete consisting only of short LTRs. However, solitary LTR can control and impact the transcription of neighbor genes or other adjacent genomic elements (3). A non-coding sequence present between the 5′ LTR and the *gag* gene contains a tRNA-specific primer-binding site (PBS) and prime reverse transcription (8); a polypurine tract (PPT) present between env and 3’LTR prime the (+) strand DNA synthesis (9). PBS specificity has been used in the past to classify HERVs into subfamilies. However, due to some degree of promiscuity in the use of PBS, this classification method fell apart.

New methods have been applied to overcome the historical classification of hERVs into three families, based on sequence homology with the original viral strains (10). Nowadays, 504 groups of HERVs and more than 700 thousands of HERV members have been described (11).

The accumulation of mutations from generation to generation has reduced or nearly halted the transcription, translation, and retrotranscription rates of these genomic elements, with some important exceptions (12). However, these genomic elements exert active functions in modulating genomic plasticity by actively affecting: a) genome stability, as they can affect the mobility of transposable elements or influence intra- and interchromosomal rearrangements (13, 14); b) the transcription rate of the neighbor genes, as the LTR elements can redirect RNAP2 activities (15), or their translation (16); c) the interference process, by acting as miRNA sponges (12, 17).

## Regulation of ERV Expression at Transcriptional and Post-Transcriptional Levels

**Figure 2:**
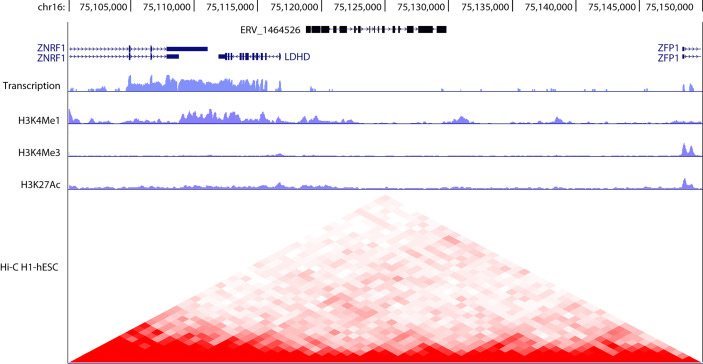
Genomic organization of hERV 1464526 in chromosome 16. Typically, as in this case, ERV elements are discontinuous and interspersed with other genomic elements. hERV_1464526 is interposed between three coding genes (ZNRF1, LDHD, ZFP1). The transcript levels as well as H3K4me1, H3K4me3, H3K27ac and Hi-C data were obtained from UCSC genome browser (https://genome.ucsc.edu/) and refer to human embryonic stem cells (hESC). A drop in the transcriptional activity is observed in correspondence to hERV_1464526 locus, in respect to neighbor coding genes. However, the heterochromatinization of ERV locus is not complete, as observed by H3K4me1 levels. Hi-C data evidence the involvement of ERV locus in chromatin looping dynamics and plasticity.

HERV are normally kept silenced by a strong epigenetic mechanism that acts at DNA (CpG methylation) and chromatin (histone methylation and heterochromatinization) levels (3) (**Figure 2**). Starting from the first, it has been proposed that the primary function of cytosine methylation might be the genome defense against transposons and retroviruses (18). Cytosine DNA methylation is a highly conserved form of epigenetic regulation among all eukaryotes. When the methylome of primary fibroblasts from seven vertebrates (human, mouse, rabbit, dog, cattle, pig, and chicken) was compared, chicken were found to have lower levels of methylation compared with mammals (19). However, the treatment with the DNA demethylating agent, 5-azacytidine (5-aza) leads to a strong de-repression of ERVs in all the examined species, including the hypomethylated chicken fibroblasts, thus confirming that DNA methylation is a conserved mechanism for repressing ERVs in vertebrates (19). The de-repression of ERVs achieved after 5-aza treatment was observed in many different physiological and pathological contexts (20–26). As a matter of fact, female mice deficient for the DNA demethylase Tet1 experience premature infertility consistent with premature ovarian failure that correlates with Line 1 and ERV activation (27).

Evolutionarily, the controlled demethylation of ERV loci has been used to regulate functional physiological processes, such as placenta formation (28). Indeed, the fusion between trophoblast cells into cytotrophoblast and then into syncytiotrophoblast during placenta formation requires a functional Syncitin-1 gene expression (29). Syncytin-1 is the envelope gene of the human Endogenous Retrovirus ERVW-1 (29). Interestingly, aberrant methylation of ERVW-1 and perturbed expression of Syncytin-1 were observed in many pregnancy diseases associated to placental morphology alterations (30). This process demonstrates that the primary methylation response after retroviral elements integration in mammalian genome during evolution underwent a process of controlled demethylation to mediate the acquisition of a new biological function (31).

Elsewhere in mammalian genome, the silencing response induced by DNA methylation of ERV elements is reinforced by the KAP1/SETDB1/DNMT1 dependent H3K9 tri-methylation (32, 33). In murine embryonic stem cells the H3K9me3 induced heterochromatinization seem to require the ATRX and DAXX-dependent deposition of histone H3.3, as H3.3 deletion decreases the H3K9me3 levels especially in correspondence to class I and class II ERVs (21, 34). The incorporation of H3.3 by the histone chaperone Daxx requires a functional Morc3-ATPase cycle and Morc3-SUMOylation, as Morc3 knock-out leads to reduced H3.3 and H3K9me3 deposition and to the de-repression of ERVs (35).

Histone citrullination (H3R8cit) has been shown to attenuate HP1α binding to H3K9me3, leading to HERV de-repression in peripheral blood mononuclear cells (PBMCs) obtained from patients with multiple sclerosis (MS) (36). Importantly, inhibition of peptidylarginine deiminase 4 (PADI4), the major enzyme responsible for the conversion of arginine to citrulline, reversed ERVs de-repression and blocked the release of inflammatory cytokines (36). However, it must be kept in mind that direct protein citrullination, and in particular citrullination of myelin basic protein, plays a controversial role in the pathogenesis of MS (37, 38).

Histone acetylation of ERVs loci should theoretically de-repress their transcription. However, conflicting data have been reported in this regard by using Histone Deacetylase (HDAC) inhibitors (39, 40). DNA methylation and histone deacetylation may act as integrated mechanisms to ensure a concerted repression of latent retroviral elements. This is supported by the finding that combined treatments of different cancer cell lines with DNMT and HDAC inhibitors strongly stimulate HERVs transcription and show synergistic effects compared with single treatments (41, 42).

In B and T cells of C57BL/6 J, DNA methylation levels in TEs of retroviral origin correlate inversely with CTCF binding. This indicates that there is a close relationship between DNA methylation and the regulation of chromatin status and loop formation (43) (**Figure 2**).

At post-transcriptional level, literature reports scattered information about a possible role of RNA interference as a mechanism to impair the stability of ERV RNAs. 19 miRNAs were found to be significantly homologous to HERV-W family members (44). Intriguingly, it has been reported that the Xist-mediated silencing of the second female X chromosome could be evolved by pretending a viral infection, as Xist sequence is recognized by the RNA binding protein Spen that has the capability to bind also retroviral RNAs (45). If confirmed, this fascinating hypothesis would explain the origin of the RNA interference mechanism as a putative ancient antiviral mechanism.

Post-transcriptional editing of ERVs RNAs has been observed in many cellular models. By performing a CRISPR screen for IAP suppressors in murine ES cells, Chelmicki and colleagues identified m6A RNA methylation as a predominant way to decrease ERV RNA stability in conditions in which their expression is re-activated (46). The methyltransferase-like METTL3 and METTL14 are two key components of the enzymatic complex that provides methylation at the LTR and 5’UTR of IAP-related ERVK elements, while the YTH-domain containing proteins act as m6A readers and triggers methylated RNA decay, which occurs probably in P-bodies (46). It was proposed that m6A-dependent ERVs regulation could decrease their immunogenic potential and the activation of inflammatory processes (46).

Adenosine-to-inosine (A-to-I) editing by ADAR (adenosine deaminase acting on RNA) of dsRNAs of retroviral origin induces mismatches in their transcripts in this way preventing their recognition by MDA5 and the activation of the inflammatory pathway (47–49). Interestingly, *Adar1^-/-^* tumors are sensitized to anti-tumor immunity and IFNβ and γ treatment (48); moreover, *Adar1* depletion allows to overcome different mechanisms of acquired resistance to immunotherapy with a significant increase in immune cells infiltration, including non-MHC I-restricted cytotoxic cells (48).

Finally, the acquisition of a Z-RNA structure by dsRNA ERV elements was proposed but only indirectly demonstrated recently (50). In particular, it was observed that murine dsRNAs of retroviral origin are recognized by the Zα-domain of Z-DNA-binding protein 1 (ZBP1) and contribute to trigger a RIPK3-dependent necroptotic process that leads to inflammation and eventually to the development of chronic inflammatory conditions as observed in RIPK1 mutated mice (50).

By analyzing the chromatin accessibility and the transcription rate of murine HSCs exposed to 5-FU treatment *in vivo*, it was observed that some transposable elements mainly belonging to the LINE1, ERV1 and ERVK families were derepressed and activate the RNA sensor MDA5 (51). This leads to the phosphorylation and activation of IRF3 and to the subsequent activation of interferon and inflammation. As a consequence of the activation of a pro-inflammatory state, these HSCs exposed to chemotherapy became more active and allowed the rapid reconstitution of murine blood system; on the opposite *Mda5 -/-* HSC failed to detect ERVs and maintained a prolonged quiescence state that allows for better long-term bone marrow performance but worse rapid response to stressful conditions such as serial 5-FU treatment (51). Similarly, ionizing radiations trigger monocyte to macrophage differentiation as a result of the establishment of a pro-inflammatory environment which is due to the significant de-repression of ERVs and their asymmetric and anti-sense privileged transcription and the subsequent activation of the MDA5/MAVS (52). This leads to the polarization of the macrophages toward a pro-inflammatory phenotype that allows their high resistance to radiation, but also the acquisition of oncogenic features (52). Despite the multiple evidence for an effect of genotoxic agents (51, 52) and radiotherapy (53) in activating the transcription of ERVs, the epigenetic mechanism underlying this phenomenon remains to be elucidated. However, the involvement of ATM kinase is highly plausible (53). Indeed, ATM has been shown to phosphorylate KRAB-associated protein-1 (KAP-1) at Ser 824 in the presence of double-strand breaks, thereby abolishing its repressive properties (54).

## dsRNA Detection: Specific Recognition and Signal Transduction

The major sources of intracellular dsRNAs are: the mitochondrial transcripts, repetitive nuclear sequences, ERVs and natural antisense transcripts (55). Mitochondrial DNA is bidirectionally transcribed and encodes 13 genes. The heavy strand encodes 12 of them, whereas the light chain encodes the thirteenth (55). dsRNAs resulting from bidirectional transcription are minimized by the action of polynucleotide phosphorylase (PNPase) and helicase HSuv3, which form a 330-kDa heteropentamer complex involved in light chain degradation (56). Defects in the mitochondrial RNA turnover pathway lead to the accumulation of dsRNAs (57) and eventually to an altered immune response as a consequence of the release of mitochondrial contents following membrane permeabilization (58). Importantly, mitochondrial dsRNAs are not substrates of ADAR (59) and if released in the cytoplasm they are preferentially recognized by PKR (55).

Nearly 70% of cellular dsRNA is the result of the transcription of repetitive elements, mainly LINEs (3%) and SINEs (67%). Although they undergo extensive adenosine deamination (60) and degradation by RNAseIII (61), untargeted dsRNAs lead to the engagement and activation of MDA5 (melanoma differentiation-associated gene 5), in the case of LINEs, and PKR (protein kinase R) for SINEs, ultimately leading to a Type I Interferon response (62).

dsRNAs also arise from antisense transcripts that pair with sense targets in the cytoplasm (55). However, the high processing rate during the interference process makes quantification and detection of sense:antisense dsRNA challenging (63).

ERVs transcripts fold into dsRNA by bidirectional transcription from LTRs or by self-paring between duplicated ERVs (41). They are frequently deaminated and detected by RLRs (55).

Regardless of their origin, cytoplasmic dsRNAs are recognized by dsRNA-binding proteins, including RIG-I-like receptors (RLRs), PKR, ADAR and oligo adenylate synthetase (OAS) (**Table 1**). Despite a certain degree of heterogeneity, dsRNA-binding proteins share a dsRNA-binding domain (dsRBD) that adopts an α−β−β−β−α topology structure close to the RNA-recognition motif (RRM) (64).

**Table 1:** Indication of the major endogenous sources of dsRNA, their privileged dsRNA-binding proteins, and their propensity to be deaminated.

*Endogenous dsRNA source*	*dsRNA binding proteins engaged*	*ADAR substrates*
*Mitochondrial dsRNA*	PKR	No
*LINE dsRNA*	MDA5	Yes
*SINE dsRNA*	PKR	Yes
*ERV dsRNA*	MDA5 > RIG-I	Yes
*ncRNA dsRNA*	Drosha, Dicer, miRISC	No

RLRs are described in detail in the following paragraph. PKR is interferon-inducible, recognizes dsRNA comprised between 33 and 85-100 base pairs (bp) in length and once activated homodimerizes and phosphorylates eukaryotic translation initiation factor 2A (EIF2A) leading to translational inhibition (65) and eventually to apoptosis (66).

ADAR enzymes (ADAR1, ADAR2, ADAR3) perform adenosine-to-inosine (A-to-I) RNA editing of dsRNAs, resulting in A:U->I:C mismatching (59). Mitochondrial dsRNAs and dsRNA derived from sense:antisense pairing are not bound by ADAR enzymes and therefore are not actively deaminated (55). dsRNA ADAR are potent dsRNA binders that sequester dsRNA and prevent their recognition by RLRs (67); at the same time, deamination has destabilizing effects on dsRNA, resulting in decreased activation of the interferon signaling pathway (68). Interestingly, Adar1 mutant mice are embryonically lethal and show and enhanced IFN signature, but concomitant deletion of the RLR MDA5 rescues the phenotype (67), whereas deletion of MAVS delays lethality (69) and RIG-I has no relevant effects (69). For completeness, we report here that it has recently been shown that transcription of ERVs can also be repressed by their genomic deamination by the cytidine deaminase APOBEC3 (70).

In humans, the OAS family consists of four genes (OAS1, OAS2, OAS3, OASL). With the exception of OASL, which is catalytically inactive, OAS enzymes bind short dsRNAs (<20 bp) and catalyze the non-processive synthesis of 2′-5′-linked oligoadenylate (2–5A) molecules which lead to the activation of endoribonuclease L (RNase L) and the subsequent degradation of the modified RNAs (71, 72).

OAS proteins, particularly OAS1, and the editing enzymes ADAR and APOBEC were found upregulated in Systemic Lupus Erythematosus (SLE) (73, 74). Conversely, mutations in *ADAR*, the gene encoding ADAR1, are associated with immune diseases, like Aicardi-Goutières syndrome (AGS), as a consequence of Type I Interferon hyperactivation (75). Similarly, the risk allele rs10774671 affecting the splicing of OAS1 is associated to the autoimmune disease Sjögren’s syndrome (76). A possible explanation for these divergent findings comes from the observation that the excessive RNA editing observed in SLE facilitates the generation of autoantigens in peripheral tissues, leading to T cells hyper-reactivity (74).

These data indicate that the balance between the decoding of dsRNA and their degradation or modification allows fine activation of the interferon pathway and maintenance of optimal levels of innate cellular immunity (55). This condition is required for the maintenance of cellular and tissue fitness.

## ERVs Intracellular Sensing and Signaling

Toll-like receptors (TLRs), in particular TLR3, are localized in the endosomal membrane and are involved in recognizing extracellular dsRNA (77). For the detection of intracellular foreign nucleic acids, cells have evolved other classes of cytoplasmic pattern recognition receptors (PRR). The cytosolic DNA sensor cyclic GMP–AMP synthase (cGAS) allows the detection of cytoplasmic DNA, while RLRs are cytoplasmic sensors of dsRNAs of retroviral origin (78). However, recent evidence prove that these two responses can be integrated. In particular, cytosolic DNA scars arising from combined radiotherapy and ATR inhibition (79) can be transcribed by RNA polIII and fold as dsRNA (80) that are recognized by RIG-I, leading to STAT1 activation and reinforcing the cGAS/STING dependent inflammatory response (79). On the opposite, reverse transcription of RNA viral genome can result in the host in the generation of dsDNA that lead to cGAS/STING activation, as reviewed in (81). An intriguing hypothesis emerging from recent literature is that activation of cGAS/STING in the presence of dsRNA is required to regulate cap-dependent mRNA translation (82) by acting at the level of PKR-like ER kinase (PERK) upstream of TBK1 to redirect ribosomes toward encoding pro-inflammatory factors (83).

RLRs family includes three members: RIG-I (retinoic acid- inducible gene 1, also known as DDX58), MDA5 (melanoma differentiation- associated protein 5, also known as IFIH1), LGP2 (laboratory of genetics and physiology 2) (78). LGP2 is structurally different from MDA5 and RIG-I and it is considered as an adaptor protein involved in the regulation of RIG-I and MDA5 functions. In particular LGP2 promotes the nucleation and the following activation of MDA5 (84), while it seems to block directly the activation of RIG-I by inhibiting its ubiquitylation (85), or indirectly through the competitive binding to dsRNAs (86) or MAVS (87). However, conventional dendritic cells (cDCs) obtained from *Lgp2-/-* or with a point mutation in the LGP2 helicase domain (K30A) were reported to be unable to release IFNβ in response to infection with RNA viruses (picornaviridae, EMCV, and mengovirus), with the exception of influenza virus, while they efficiently responded to synthetic poly I:C (88). Therefore, further studies seem necessary to clarify the role of the CARD-less LGP2 in regulating MDA5 and RIG-I signaling.

RLRs have a central SF2 helicase domain with ATPase activity and a carboxy-terminal domain (CTD) that bears an RNA binding domain. While in RIG-I the binding to RNA leads to the unlock of its closed conformation and to the formation of short RIG-I oligomers, in MDA5 it triggers the polymerization of long MDA5 filaments (89). This difference reflects their binding preference as RIG-I binds 5’-triphosphate ssRNA (90) and small dsRNAs (70-2000 bp long, like the ones generated by negative strand paramyxoviruses, influenza virus and Japanese encephalitis virus), whereas MDA5 recognizes long dsRNA molecules, as the positive strand picornaviruses (> 2kb long) (91, 92). In fact, *Rig1-/-* mice are embryonic lethal, while *Mda5 -/-* mice are healthy up to 6 months of age, but in both the cases the knock-out mice are highly sensitive to viral infections with the respectively recognized viral strains (91). RIG-I and MDA5, but not LGP2, contain two caspase activation and recruitment domains (CARDs) at their amino terminus. Once activated, as a consequence of a conformational change, MDA5 and RIG-I expose the CARD domains. This allows homotypic CARD–CARD interactions with MAVS. MAVS (93–95) is a mitochondrial protein that once activated by RLRs gives rise to the formation of fibrils with a prion-like structure (96) in response to a TRIM31-dependent K63-linked MAVS polyubiquitination; once aggregated, MAVS activates TBK1 and IKKϵ kinases, which in turn activate the transcription factors IRF3/IRF7 and NF-κB, ultimately leading to type I interferon pathway activation (97). The deletion of the CARD domain at the N-terminus of MAVS prevents its RLR-activated aggregation (98) and displays a dominant-negative effect on endogenous MAVS aggregation and signaling (94). Similarly, IFNAR depletion or IFN signaling blockage blunts RLR/MAVS pathway and limits the effects of cytolysis observable after an RNA virus infection (99, 100).

The activities of RLRs are deeply regulated at post-transcriptional levels (78). RIG-I is activated by the TRIM25 mediated K63 poly-ubiquitylation of its CARD domain that triggers RIG-I oligomerization (101). Conversely, the K48 poly-ubiquitylation of RIG-I triggers its proteasomal degradation (102), that is blocked by its TRIM38 dependent SUMOylation (103). The phosphorylation of RLRs is linked to their inactivation: the PKC and CKII mediated phosphorylation of RIG-I (S8, T170, T770, S854, S855) and the RIOK3 mediated phosphorylation of MDA5 (S88, S828) are both reversed by the activating dephosphorylation exerted by PP1 (78). The acetylation of RIG-I in its CTD blocks its ability to bind dsRNA, while its HDAC6-mediated deacetylation switches on its activity (104). Curiously, another class II HDAC, HDAC4, was identified for its ability to block the antiviral response by inhibiting IRF3, TBK1 and IKKϵ downstream to PRR (105), or by regulating the acetylation status of well-defined inflammatory -related super-enhancers (106). Finally, many partners regulate the oligomerization of RIG-I and MDA5 or act as co-receptors of dsRNAs (ZCCHC3, DDX60, DHX15, PACT, reviewed in (78)), while 14-3-3 proteins mediate the cytosol-to-mitochondria translocation of RLRs, thus allowing their interaction with MAVS (107).

## Impact of RLRs on Cellular Senescence and Aging

RLR signaling was reported to impact on cellular processes likewise cellular senescence, proliferation and survival, and on physiological and pathological processes such as aging and autoimmune diseases (108–110).

Aged senescence-accelerated mouse prone-8 (SAMP8) mice are characterized by the activation of RIG-I/NF-κB pathway and the accumulation of proinflammatory cytokines (IL-6, NO) in the absence of exogenous viral stimulation (111). Moreover, RIG-I was found to be up-regulated both at RNA and protein levels in models of replicative senescence and murine aging, in an ATM-dependent manner (112). In senescent cells, RLRs activation promotes TBK1-dependent phosphorylation of IRF3 and IRF7 (primary response), their subsequent nuclear translocation and the transcription of IFN and IFN-regulated transcription factors (secondary response) that further reinforce IFN-signaling (113). In parallel, RLRs trough MAVS engagement trigger the NF-κB dependent induction of IL-6 and IL-8, while Klotho suppresses RIG-I-mediated senescence-associated inflammation by directly interacting with it and blocking its multimerization (112). On the contrary, *Rig-I^-/-^* mice display marks of premature aging like hair loss and moderate lethargy and *Rig-I^-/-^* MEFs fibroblasts undergo a premature replicative senescence characterized by amplified integrin β3/p38 MAPK signaling (114). These two reported functions of RIG-I are difficult to reconcile unless we hypothesize that the existence of compensatory circuits, such as the one involving MDA5, may affect the phenotype observed in *Rig-I^-/-^* mice.

Indirect evidences link MAVS to the induction of cellular senescence. In SLE, Bone marrow-derived mesenchymal stem cells (BM-MSCs) undergo premature senescence that correlates with the increased mRNA and protein levels of MAVS and IFNβ (115). The knock-down of MAVS partially rescues the phenotype by decreasing the levels of p16 and p53 and attenuating the release of pro-inflammatory cytokines (115).

The unclear role of RLRs in affecting senescence onset is traced by the reciprocal influences of the oncogene HRAS on RLR signaling. In fact, while oncogenic HRAS (*HRAS^G12V^*) strongly inhibits Sendai virus-induced type I interferon signaling, ectopic expression of HRAS or the catalytically inactive HRAS^N17^ in 293 cells impairs the formation of MAVS-TNF receptor-associated factor 3 signaling complexes (116).

In consideration of these data, it is even more urgent to determine the impact of RLRs modulation in models of Oncogene-Induced senescence.

Patients with mutations in RIG-I and MDA5 suffer for autoimmune diseases. RIG-I mutations lead to atypical Singleton-Merten Syndrome, while MDA5 mutations are associated to classical Singleton-Merten Syndrome, Aicardi-Goutières syndrome, Systemic Lupus Erythematosus, Type 1 Diabetes and Graves disease (117). The RLR missense mutations observed in these diseases are gain-of-function and lead to exaggerated Type I Interferon response activation (118), probably reinforced by autoantigens generation (74).

How much RLRs over-activation depends on de-repression of ERVs is still unknown. However, it is not incorrect to hypothesize that a prolonged basal RLR activation following a prolonged exposure to RNA viruses or to ERVs de-repression could lead to the establishment of chronic inflammatory states.

## ERVs in Cellular Senescence, Neurodegeneration and Aging

**Figure 3:**
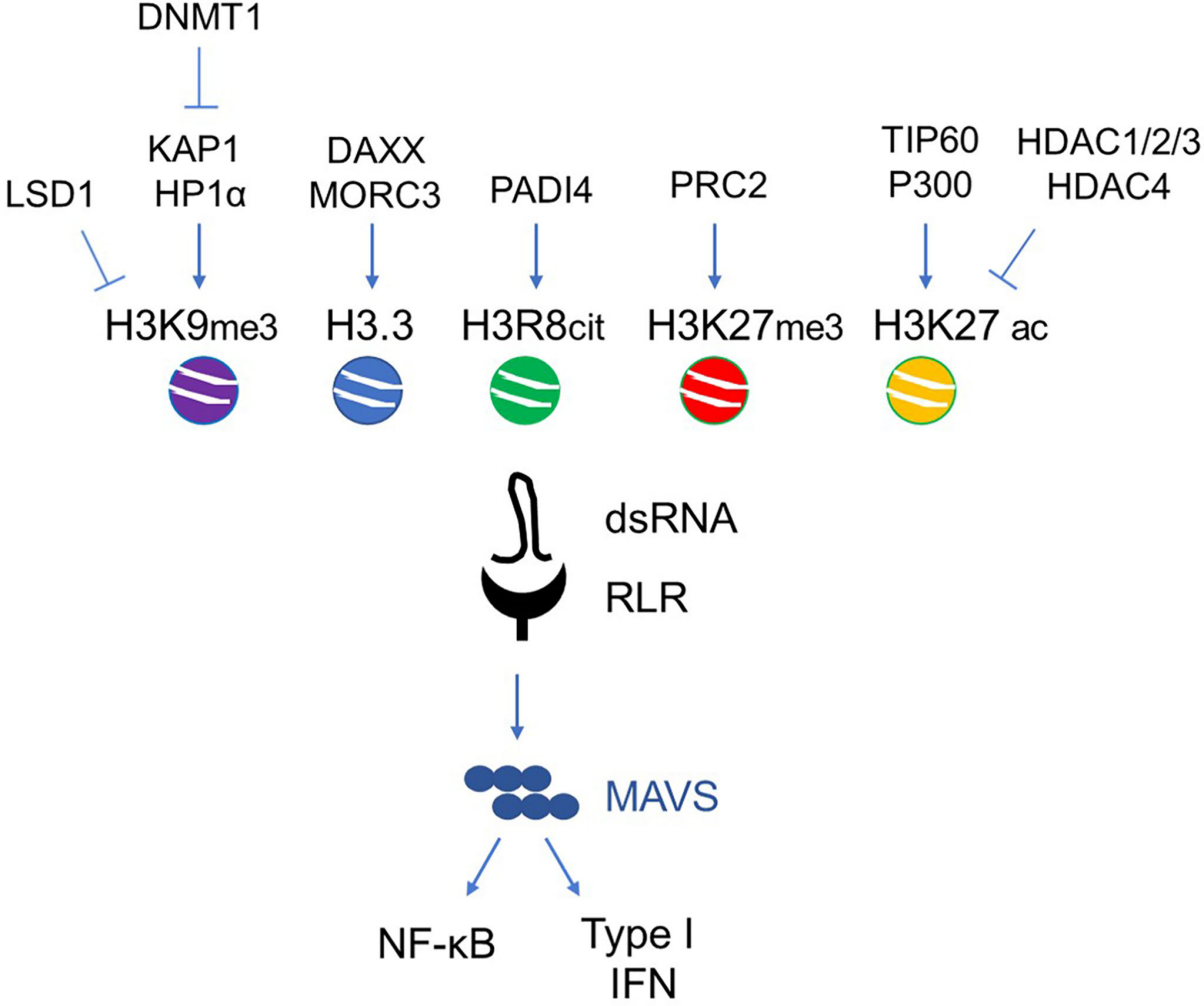
Main histone epigenetic regulators involved in ERV de-repression during senescence and aging. Exemplary scheme of the agonistic/antagonistic epigenetic regulators acting on the chromatin remodeling of ERV loci.

A deep epigenetic resetting (119) and the activation of certain super-enhancers (106) characterize replicative senescence and aging. On the opposite, heterochromatinization has been considered for decades a typical feature of senescent cells (120), even though by the time its specificity and relevance as senescent marker has been a matter of debate (121, 122). In general, a perturbation of the epigenetic environment has been reported to trigger or allow the escape of cellular senescence, demonstrating in this way the centrality of the epigenetic control of this process (119). The alteration of heterochromatin/euchromatin compartments lead to the activation of TE and ERVs expression in different models of cellular senescence (123) (**Figure 3**). ERV expression is expected to sustain the establishment of a senescence-associated secretory phenotype (SASP) (123), which in turn should reinforce the senescence response (119, 124). Accordingly to this evidence, the PRC2-dependent heterochromatinization induced by re-expressing the methyltransferase DNMT3L in pre-senescent MEFs allows the repression of ERVs and a marked delay in the onset of senescence (125).

Methyl-binding domain sequencing and RNA sequencing of murine skeletal muscles evidenced a global down-regulation coupled to increased DNA methylation of ERVs in the early phase of aging, that is followed by their de-repression and demethylation in the late phase (>20 months) of aging (126). Curiously this methylation rewiring was not observed in T cells (126).

By comparing the expression of HERV-K and HERV-W proviruses in red blood cells obtained from young and nonagerians individuals, Nevalainen and colleagues observed a statistically significant differential expression among the two groups of 3 out of 33 and 1 out of 45 detectable HERV-K and HERV-W members (127). Despite the slightest differences, the global expression of the HERV-K and HERV-W members allows the two groups to be perfectly segregated (127). On the contrary, by analyzing by means of qPCR the transcriptional activity of HERV-H, HERV-K, HERV-W, and HERV-E in PBMCs from 261 subjects, Balestrieri and colleagues identified increased levels of HERV-K and HERV-W in oldest subjects (128). Similarly, in another study, the DNA methylation status of HERV-K loci in 177 samples of peripheral blood mononuclear cells obtained from volunteers between 20 and 88 years of age evidenced two waves of demethylation that occur during ages 40-63 and again during ages 64-83 (129). To explain at least in part the ambiguities and contradictions associated with the expression of ERVs during aging, it has been demonstrated that the percentage of circulating senescent human hematopoietic stem and progenitor cells (HSPC) does not increase with age, as they are actively cleared by the organism as a consequence of the release of eat-me signals (130). This evidence is questioned by the concept of immunosenescence, that will be explored later on in the manuscript. Furthermore, due to technical difficulties, it is currently not possible to clarify how many of the transcripts resulting from ERV de-repression are actively deaminated and inactivated (22).

On the opposite, transposable elements (TE), and ERV in particular, were de-repressed in HSPC cells brought to senescence *in vitro* (130). This de-repression is coupled to DNA demethylation that occurs in senescent HSPC cells (131). In particular, hypomethylated regions in senescent HSPC cells obtained from three healthy donors and sorted out on the basis of senescence-associated beta-galactosidase (SA-βgal) activity (C12FDG staining) are focal rather than global (131) and mainly fall in correspondence to repetitive elements and active enhancers characterized by CEBP binding (131). Curiously, expression of repetitive elements (Alu, ERV1, ERVL, ERVK, and LTR retrotransposons) and inflammatory cytokines is attenuated in leukemic stem cells (LSCs) in acute myeloid leukemia (AML), suggesting that escape from senescence and immune surveillance may be the origin of AML (132).

Tauopathies are neurodegenerative diseases characterized by the accumulation of pathological deposits of Tau protein in the brain (133). In Alzheimer’s disease (AD), the accumulation of misfolded amyloid-β peptide or of Tau aggregates leads to heterochromatin relaxation and de-repression of silenced loci that ultimately leads to DNA damage and activation of cellular inflammation (134). De-repression of retroviral and transposable elements have been proposed to play a role in neurodegeneration and AD (3, 135). Retrospective analysis of 636 deceased AD-affected-subjects identified significant de-repression of HERV-Fc1 transcripts (136). In a *Drosophila* model of AD achieved by expressing a mutant isoform of Tau protein (tau^R406W^) and analyzing the data obtained from head samples at day 10 of adulthood, an age at which neuronal deficits become evident, the same authors identified a significant de-repression of retroviral elements, similarly to what observed in human samples (136). RNA-seq analysis performed in tau^R406W^ and WT heads identified 50 and 60 transposable elements that are respectively significantly increased and decreased in tau transgenic model (137). Most of them are Class I long terminal repeat (LTR) retrotransposons (137). Moreover, the forced mobilization of transposable element achieved in tau^R406W^ flies by silencing the flamenco locus increased neurotoxicity and locomotor deficits. In tauopathy, the mobilization and transcription of transposable elements is the result of the decreased expression of piwi and piRNAs (137) and heterochromatin decondensation (137), probably as a result of impaired H3.3 turnover (138).

Curiously, the de-repression of ERVs was observed also in cancer models and in cancer patients (139). In tumors, TERT promotes the Sp1-dependent de-repression of hERVs that fold in dsRNA and trigger the RIG-I/MDA5-MAVS signaling thus inducing inflammation and creating an immunosuppressive environment (140). The de-repression of retroviral elements was observed also in melanoma patients and was the result of CpG demethylation (141). Decreased DNA methylation levels allow the segregation between nevi and malignant melanocytic lesions and successful predict a worse prognosis (141). It has been reported that the activation of p53 leads to the repression of the H3K4 Histone demethylase KDM1A (LSD1) and the DNA demethylase DNMT1 and to the subsequent de-repression of hERV transcription. The concomitant repression of RISC components DICER, AGO2, and TRBP2 and the induction of dsRNA sensors RIG-I and MDA5 lead to the activation of Type I and III Interferon responses and could be exploited to overcome cancer resistance to immune checkpoint blockage (142). In multiple myeloma (MM), the dual inhibition of H3K27 and H3K9 methyltransferases EZH2 and G9a leads to the de-repression of ERVs genes, the activation of IFN signaling, the suppression of IRF4-MYC axis and the impairment of xenograft formation by MM cells in mice (143). Finally, radiation resistant head and neck squamous cell carcinoma clones are characterized by the acquisition of a senescence-associated secretory phenotype (SASP) and the de-repression of ERVs (ERV3-1) (144).

To sum up, ERVs were found to be reactivated and actively transcribed in patients with autoimmune diseases, neurodegeneration, cancer (139, 145, 146) and during physiological aging (123, 127, 131, 147). Common characteristics of these states are the accumulation of DNA damage, ER-stress and higher systemic inflammation (148). Different cellular responses are mediated by the activation of interferon in these states: survival, cell death, cytokines release. More work is required to clarify whether these differential responses depend on the intensity and magnitude of the signaling pathways activated or on the differential signal transducers activated in the different contexts. Moreover, the integration of the RLR and cGAS/STING pathways (79, 80) makes the analysis of the contribution of the different signaling molecules even more complex but reiterates the importance of nucleic acid sensors in regulating not only cellular innate immunity but also cellular fitness (83).

## ERVs and RLRs in Immunosenescence

Immunosenescence is characterized by increased proportions of CD14+ monocytes, decreased CD19+ B lymphocytes and increased proportions of CD4+CD28- and CD8+CD28- T lymphocytes, resulting in a deep immunosuppression concomitant with an high inflammation (149). The aging-associated decline of the immune system was observed to predispose individuals to fatal infections or increase the risk of cancer (150). By comparing the expression levels of *HERV-K(HML-2)* in the peripheral blood mononuclear cells between nonagenarians (n=61) and young controls (n=37), Marttila and colleagues identified lower levels of *HERV-K(HML-2)* in nonagerians and *HERV-K(HML-2)* levels did not correlate with any marker of immunosenescence (151). Moreover, the release in the blood of pro-inflammatory cytokines (TNF-α, IL-1β, IL-6, and IL-8) after stimulation with RLR ligands (5’ppp-dsDNA and poly(I:C)) was comparable between young (20-39 years) and elderly (60-84 years) healthy participants (152). On the contrary, an indirect evidence of a driving role of HERV de-repression in inflammaging comes from the analysis of HERV-K (HML-2) provirus levels in the peripheral blood mononuclear cells (PBMC). In PBMC obtained from older individuals, HERV-K transcript levels correlate with deregulated activities of neutrophils (147).

Psoriasis is a Th17 cytokine-mediated chronic inflammatory disease; CD8+ T cells obtained from psoriasis patients display features of immunosenescence as they are for the most part senescent or terminally differentiated. Curiously, psoriasis patients have increased proportions of Tregs but with a decreased regulatory potential (153). Interestingly, MDA5 levels are increased in psoriatic lesions. Whether this represents a consequence of the inflammatory state or its source is still unknown (153).

Monocytes obtained from older (age 65-89) adult donors in respect to younger (age 21-30) donors were found to be impaired in IFN induction after exposure to RIG-I–specific 5’-ppp 14-bp dsRNA ligand (113), correlating to the increased vulnerability of elderly people to Influenza A virus (IAV) infection (154). The impairment in RIG-I signaling in aged monocytes is related to the increased basal proteasomal degradation of the adaptor protein tumor necrosis factor receptor–associated factor 3 (TRAF3) and to the defective induction of IRF8, as the re-expression of TRAF3 and IRF7 rescues IFN-signaling (113). Defective IFN signaling was detected also in Plasmacytoid Dendritic Cells (pDCs) from elderly people after the engagement of TLR (155) and in memory T cells after TCR engagement (156).

Further studies are needed to determine whether ERV de-repression may directly lead to immunosenescence or is merely a contributory cause that combines with the signaling decoding defects observed in aged immune cells. A better understanding of the multiple defects in innate antiviral signaling that occur with aging will help identify new potential targets for precise therapeutic interventions.

## Discussion: Does the ERV Dependent Activation of RLR (RIG-I-Like Receptors)-MAVS Pathway Directly Control Senescence and Aging?

The de-repression of ERVs and the activation of RIG-I/MDA5/MAVS pathway were observed during neurodegeneration, cancer, aging and autoimmune diseases (108, 109). Whether loss-of-function experiments have clarified the direct role of RLRs in sustaining the inflammatory states and the interferon signaling observed in this physiological and pathological states, the studies regarding the roles played by ERV in sustaining this signaling pathways are mainly correlative. The high number of repetitions and their inhomogeneous distribution in the genome make the modulation of ERV expression biotechnologically demanding. New approaches based on CRISPR/Cas9 technology are demonstrating that the expression of ERVs and the subsequent activation of RLRs can impact on cellular fitness; however, the intra-cellular and extra-cellular effects resulting in ERVs de-repression appear to be only complementary to a primary stressful event, such as DNA damage (157) or the activation of a basal inflammatory state (158). Nevertheless, RLRs inhibition holds promise for the treatment of autoimmune diseases and interferonopathies, while RLR agonists may have a future in cancer therapy, to induce a pre-senescence state or to increase sensitivity to apoptotic agents. In this regard, agonistic activation of RIG -I by IVT4 was found to increase tumor shrinkage by CD8+ and NK cells in immunocompetent EGFR-driven *in vivo* tumor models treated with EGFR inhibitors (159).

## Author Contributions

EG: conceptualization, writing, editing. LX: writing, editing. All authors contributed to the article and approved the submitted version.

## Funding

This work was supported by the grants from AIRC - Associazione Italiana per la Ricerca sul Cancro to EDG (MFAG2020 ID 25000) and LX.

## Conflict of Interest

The authors declare that the research was conducted in the absence of any commercial or financial relationships that could be construed as a potential conflict of interest.

## Publisher’s Note

All claims expressed in this article are solely those of the authors and do not necessarily represent those of their affiliated organizations, or those of the publisher, the editors and the reviewers. Any product that may be evaluated in this article, or claim that may be made by its manufacturer, is not guaranteed or endorsed by the publisher.
